# Chromoblastomycosis Caused by Fonsecaea monophora Mimicking Lichen Planus

**DOI:** 10.7759/cureus.53863

**Published:** 2024-02-08

**Authors:** Yasmine Oprea, Thomas Stringer, Daiva Mattis, Bijal Amin, Ranon Mann

**Affiliations:** 1 Dermatology, Albert Einstein College of Medicine, Bronx, USA; 2 Dermatology, Georgetown University Medical School/MedStar Washington Hospital Center, Washington, DC, USA; 3 Dermatopathology, Albert Einstein College of Medicine, Bronx, USA; 4 Dermatology, Montefiore Medical Center, New York City, USA

**Keywords:** lichen planus, fungal infection, fonsecaea monophora, dematiaceous fungi, chromoblastomycosis

## Abstract

Chromoblastomycosis is a rare fungal infection acquired by traumatic inoculation of pigmented fungi from an environmental source. The polymorphic presentation of chromoblastomycosis may mimic other dermatologic conditions, leading to delays in diagnosis. Thus, histopathology is critical in identifying the presence of fungi and confirming the diagnosis. We present a case of chromoblastomycosis caused by the organism *Fonsecaea monophora* mimicking a lesion of lichen planus to highlight the importance of histopathology in the diagnosis of this condition.

## Introduction

Chromoblastomycosis is a saprophytic fungal infection endemic to the equatorial regions of the world and is associated with occupational risk factors such as agriculture, outdoor exposure, and natural disasters [1,2]. Due to its increased prevalence in tropical and subtropical areas, few cases have been reported in North America. Of these, most have been reported in the southern region of the United States. Data suggests that the prevalence of chromoblastomycosis in the United States is estimated to be one in eight million [2]. Following traumatic inoculation of the fungi from an external environmental source, an initial lesion may appear and assume a papulosquamous, nodular, tumoral, verrucous, or cicatricial morphology. The variable presentation of chromoblastomycosis may mimic other dermatologic conditions and render it susceptible to misdiagnosis. Histopathology is critical to identify the presence of fungi in a lesion and confirm a diagnosis of chromoblastomycosis.

## Case presentation

**Figure 1:**
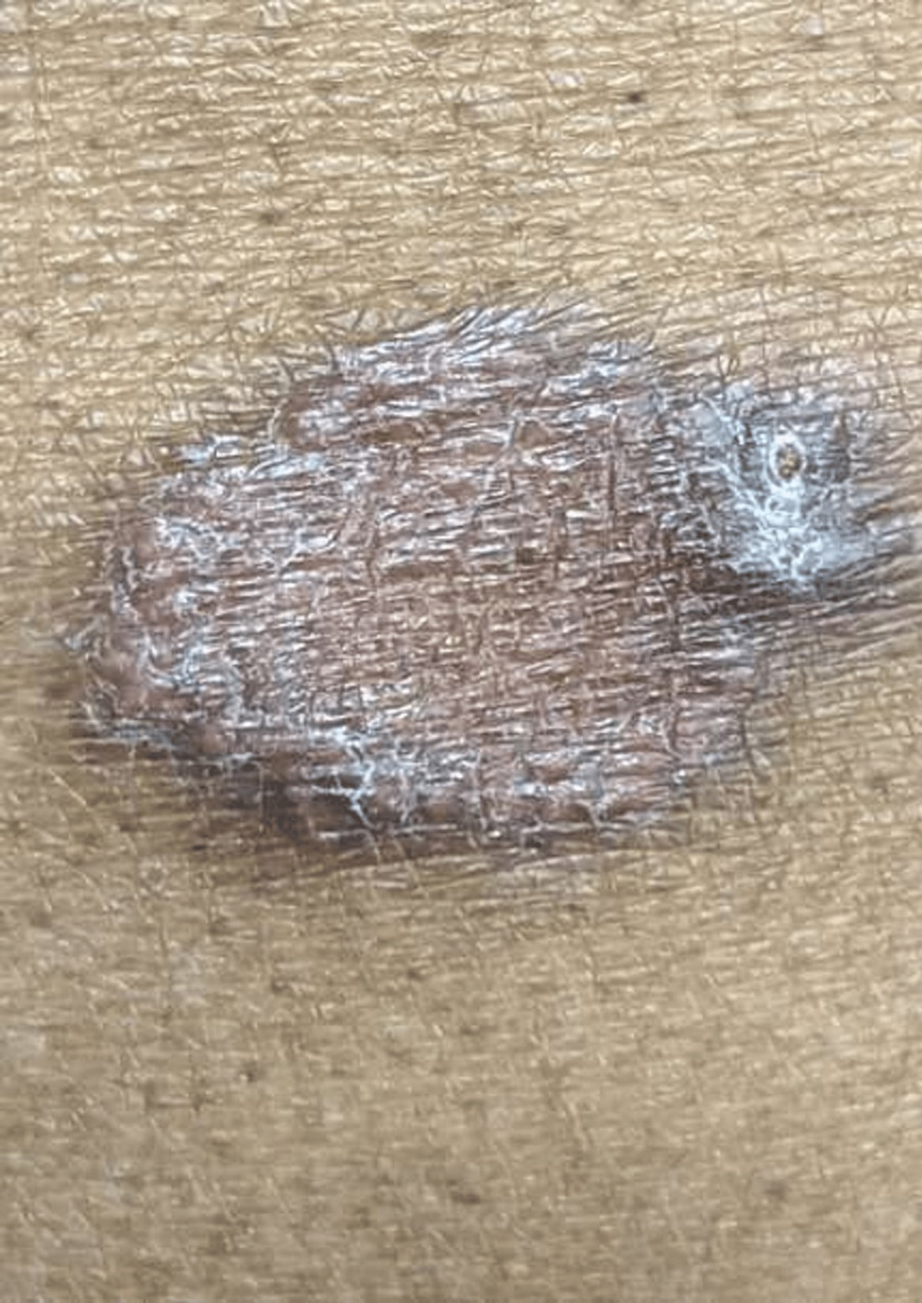
Purple-to-brown, ovoid, scaly plaque on the left upper abdomen.

A 70-year-old male with a medical history of prediabetes, hypercholesterolemia, and benign prostatic hyperplasia presented with an itchy plaque on the left upper abdomen for one year. The lesion remained stable, as the patient did not note any changes over time. The lesion had previously been treated with betamethasone dipropionate ointment for one month with no response nor any worsening upon treatment. The patient denied a personal or familial history of rashes, antecedent traumatic injury to the area, or recent travel outside of the United States. A physical examination was significant for a solitary 3-cm ovoid, purple-to-brown, well-demarcated scaly plaque with a raised border on the left upper abdomen (Figure 1). Physical exam was negative for Wickham striae on the oral mucosa. No other cutaneous abnormalities were observed. Based on the clinical presentation, a diagnosis of lichen planus was suspected.

**Figure 2:**
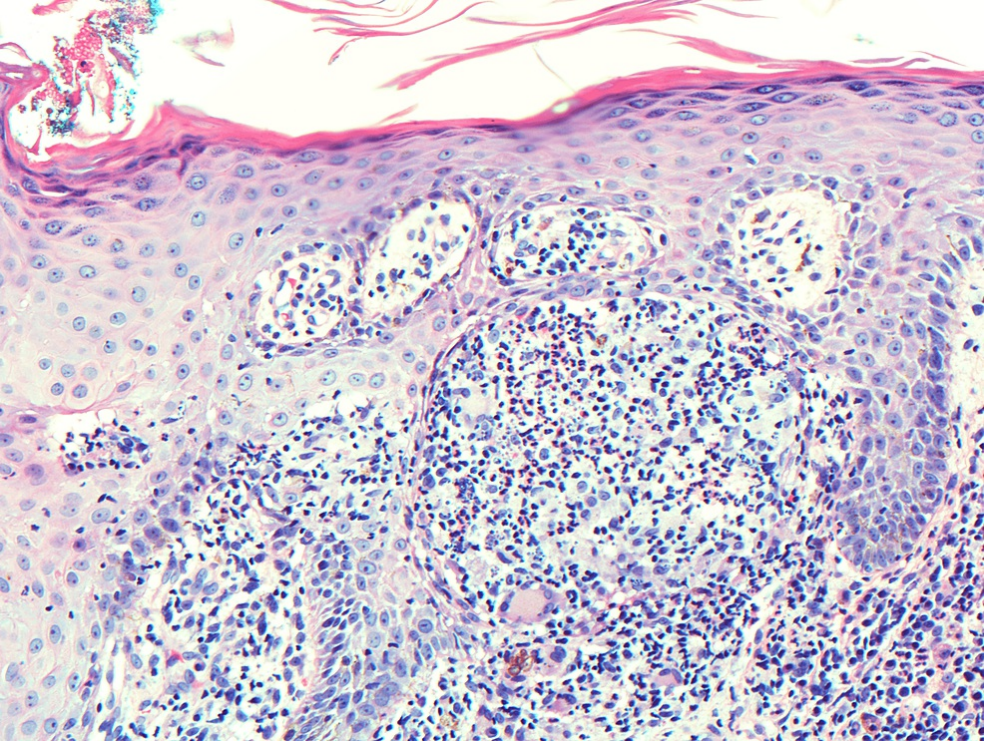
Lichenoid infiltrate in the dermis (H&E stain; original magnification ×200).

**Figure 3:**
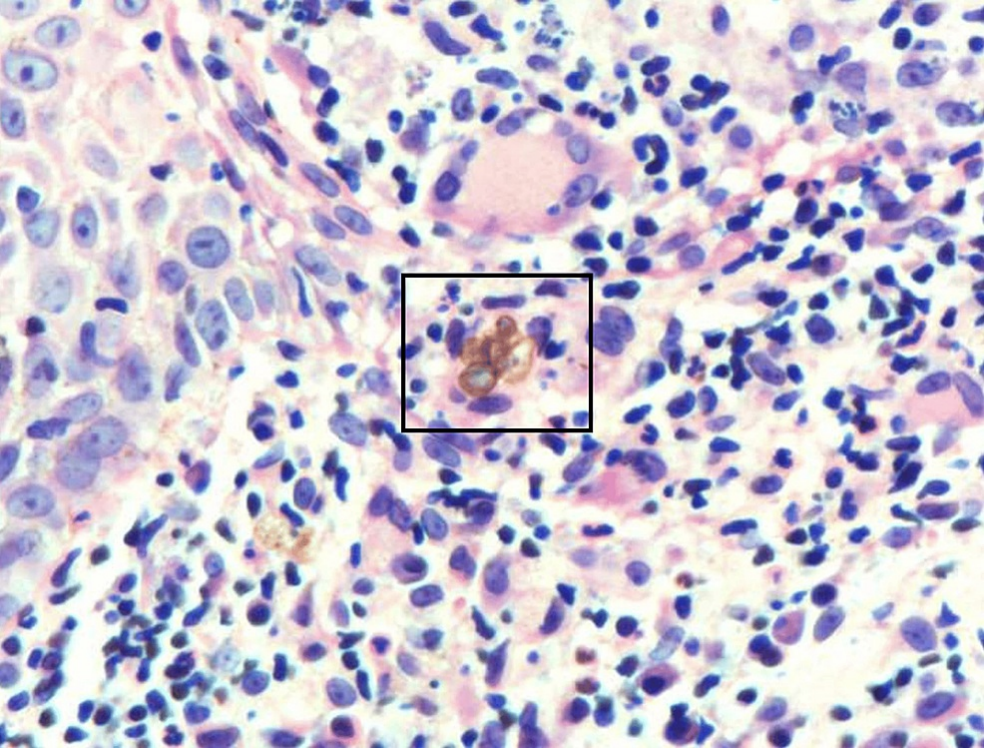
Medlar bodies within multinucleated histiocytes in the dermis (H&E stain; original magnification ×600).

An initial potassium hydroxide (KOH) preparation of the lesion was negative for hyphal elements. A punch biopsy at the periphery of the lesion was then taken, and the histopathologic examination showed a lichenoid inflammatory cell infiltrate along with the presence of giant cells (Figure 2). Deeper sections revealed a mixed cell dermal infiltrate including collections of histiocytes, lymphocytes, neutrophils, and eosinophils. Pigmented yeast (Medlar bodies) were then observed within multinucleated histiocytes (Figure 3). Periodic acid-Schiff stain highlighted fungal spores, confirming the diagnosis of chromoblastomycosis. 

Upon the patient’s return for follow-up, a repeat KOH examination was performed, and Medlar bodies were identified. A fungal culture was performed at our hospital and initially identified the organism as part of the genus Cladosporium. However, the sample was then sent for confirmation to the University of Texas at San Antonio, United States, where a PCR amplification of the internal transcribed spacer (ITS) and transcription elongation factor (TEF) 1-alpha genes revealed that the causative species was in fact *Fonsecaea monophora *(*F. monophora*). The patient underwent surgical excision of the lesion without complication and was initiated on itraconazole 100 mg twice daily for two months. The patient had no sign of recurrence when last seen sixteen months after the excision.

## Discussion

Chromoblastomycosis is an implantation mycosis caused by pigmented saprophytic fungi [1]. The organism enters through breaks in the skin barrier and then presents with skin lesions after a variable time frame, ranging from several weeks to months. Infection, which occurs in both immunocompetent and immunocompromised hosts, is more often observed in men, perhaps as a result of hormonal influence and environmental exposure. Although the most common sites of inoculation include the hands and lower extremities, infections have been reported in other areas of the body as well. Similar to the findings in this patient, a solitary pruritic plaque may be the initial presentation. However, the lesion can progress to develop satellite lesions and assume a nodular or tumoral appearance [2].

On histopathological analysis, a characteristic feature of chromoblastomycosis is the presence of Medlar bodies (also known as sclerotic or muriform cells), which are aggregations of spherical, pigmented fungal cells. Medlar bodies may be seen on both KOH preparations and histopathological sections and represent the fungus in its pathogenic state [1]. The immune response to the organism is driven predominantly by Th2+ T-helper cells and can induce a hyperproliferative cutaneous response which may lead to the development of papulosquamous, verrucous, or cicatricial lesions. The variable manifestations make chromoblastomycosis prone to misdiagnosis since the inciting traumatic incident can often be forgotten [2]. A common initial presentation of chromoblastomycosis may involve a smooth papular lesion that can progress to a papulosquamous appearance with satellite lesions [2]. As in the current case, lesions may mimic the appearance of common inflammatory dermatoses, such as lichen planus, which also has a variety of clinical presentations [3]. In this case, the patient did not recall a history of skin trauma, and the cutaneous lesion assumed the appearance of lichenoid dermatitis on both initial clinical and histopathologic evaluation. 

Due to the protean appearance of chromoblastomycosis, the differential diagnosis that may be considered is extensive. Mycoses such as coccidiomycosis, blastomycosis, and phaeohyphomycosis, as well as bacterial infections such as cutaneous tuberculosis or leprosy, might be considered. Other cutaneous conditions mimicked by chromoblastomycosis may include cutaneous leishmaniasis, psoriasis, lichen planus, and sarcoidosis [2,4]. In the case of our patient, the clinical presentation was suggestive of annular lichen planus.

Clinical complications of untreated chromoblastomycosis include fibrosis, lymphatic stasis, and secondary bacterial infections. Fibrosis and lymphatic stasis caused by contiguously and slowly progressing chromoblastomycosis may resemble lymphedema and may cause significant disability in patients [2].

Treatment options for chromoblastomycosis are dependent on the extent of the lesion. While cryotherapy or surgical excision may suffice for small lesions, the removal of larger or more numerous lesions may lead to a risk of dissemination [2]. Systemic antifungals, such as itraconazole or terbinafine, may be used either alone or in conjunction with surgical methods to increase efficacy and promote clinical improvement [2,5,6]. Itraconazole is the most commonly used oral antifungal agent to treat chromoblastomycosis, often requiring months of treatment in order for the lesions to resolve [7]. Laser therapy, which allows for precise ablation of lesions, has been utilized as well [2,8,9]. Despite several treatment methods described in the literature, current evidence supports a combination of surgical excision and systemic antifungals as optimal for providing a complete clinical cure [2,10-12].

*F. monophora*, the causative organism of our patient’s lesion, is a dematiaceous fungus found primarily in Eastern Asia and is among the causative organisms of chromoblastomycosis. A study conducted in China evaluated isolates from 24 patients diagnosed with chromoblastomycosis and revealed that 20 of those cases were caused by strains of *F. monophora, *demonstrating that *F. monophora *is the most prevalent agent of chromoblastomycosis in that region [13]. However, there may be geographic variation in the prevalence of *F. monophora*. In addition to causing cutaneous disease, *F. monophora* is known to be a neurotropic organism and has been cultured from cerebral abscesses, most commonly in immunocompromised patients [14-16]. The mechanism of extension to the brain is unknown. However, *Fonsecaea pedrosoi* is still considered to be the most common causative organism of chromoblastomycosis, accounting for approximately 90% of cases [2,4]. It is uncertain how our patient was exposed to *F. monophora.*

## Conclusions

Chromoblastomycosis, a dematiaceous fungal infection which may produce varied cutaneous morphologies, may be a diagnostic challenge for clinicians. As in the current case, lesions mimicking the clinicopathologic appearance of common inflammatory dermatoses such as lichen planus can be observed. Although uncommon, the diagnosis should be considered even in patients without a history of travel and skin trauma. To confirm the diagnosis and allow for proper management of the lesion, a KOH preparation or skin biopsy demonstrating Medlar bodies is required.
